# A Community-Based Marketing Campaign at Farmers Markets to Encourage Fruit and Vegetable Purchases in Rural Counties With High Rates of Obesity, Kentucky, 2015–2016

**DOI:** 10.5888/pcd14.170010

**Published:** 2017-08-31

**Authors:** Emily DeWitt, Margaret McGladrey, Emily Liu, Nicole Peritore, Kelly Webber, Brooke Butterworth, Ann Vail, Alison Gustafson

**Affiliations:** 1 Department of Dietetics and Human Nutrition, University of Kentucky, Lexington, Kentucky; 2 University of Kentucky College of Public Health, Lexington, Kentucky; 3 Family and Consumer Sciences Extension, University of Kentucky, Lexington, Kentucky; 4 School of Human Environmental Sciences, Family and Consumer Sciences, University of Kentucky, Lexington, Kentucky

## Abstract

Availability of farmers markets may increase fruit and vegetable consumption among rural residents of the United States. We conducted a community-based marketing campaign, Plate it Up Kentucky Proud (PIUKP), in 6 rural communities over 2 years to determine the association between exposure to the campaign and fruit and vegetable purchases, adjusted for Supplemental Nutrition Assistance Program recipient status. Logistic regression was used to examine the odds of the PIUKP campaign influencing purchases. Awareness of the PIUKP marketing campaign was significantly associated with a willingness to prepare fruits and vegetables at home. Using marketing strategies at farmers markets may be an effective way to improve fruit and vegetable purchases in rural communities.

## Objective

A diet rich in fruits and vegetables is associated with disease prevention and management (1–3), yet US adults overall do not meet the goals of Dietary Guidelines for Americans, and rural residents consume fewer fruits and vegetables relative to their urban counterparts (4). Farmers markets may improve fruit and vegetable purchasing habits (5). Understanding is limited about how to engage residents in rural areas to visit farmers markets and purchase fruits and vegetables, although transportation and cost (6) and limited cooking and nutrition knowledge (7) have been identified as barriers. We conducted a community-based marketing campaign over 2 years (September 2014–September 2016) targeting primary shoppers (shoppers who conducted at least 25% of the household shopping) at farmers markets in 6 rural counties in the Delta and Appalachian regions of Kentucky, where adult obesity rates are greater than 40%, to determine whether the campaign increased purchases of fruits and vegetables.

## Methods

The Plate it Up Kentucky Proud (PIUKP) program (https://fcs-hes.ca.uky.edu/content/plate-it-kentucky-proud) is ongoing in partnership with the University of Kentucky Department of Dietetics and Human Nutrition, the Kentucky Department of Agriculture, and the University of Kentucky Cooperative Extension Service. PIUKP used samples of healthy foods, recipe cards, radio advertising, newspaper advertising, incentives, and poster board displays to increase the purchase, preparation, and preservation (ie, canning or freezing) of Kentucky-grown food products through marketing and partnerships with educational institutions. PIUKP was conducted at 6 farmers markets in 6 rural Kentucky counties. The markets were in partnership with the University of Kentucky Cooperative Extension. County size ranged from 10,000 to 30,000 residents, and farmers markets in these counties served an average of 125 customers daily. Extension agents prepared food samples and provided recipes using local and seasonal fruits and vegetables to promote PIUKP in their communities.

Data were collected by graduate students in summer 2015 (year 1) and summer 2016 (year 2). Six farmers markets, one in each county, were visited on various days and times in July and August of both years. Students spent an average of 2.5 hours at each market. A cross-sectional survey and convenience sample were used in both years. The University of Kentucky institutional review board approved the study. Demographic questions asked about preferred language, sex, age, car ownership, education, Supplemental Nutrition Assistance Program (SNAP) recipient status, race, and income. Participants were asked to describe their eating habits over the past year, including fruit and vegetable intake, intent to purchase fruits and vegetables in relation to PIUKP, and awareness of PIUKP. Participation was voluntary, and paper-and-pencil surveys were self-administered. Farmers market patrons were asked to complete the survey regardless of whether they sampled the food prepared from the recipe. In both years participants were given a tote bag as an incentive for sampling the food and completing the survey. In year 2, to test the strategy of reducing travel cost as a barrier, $5 gas cards were also given to SNAP participants who lived more than 9 miles from the farmers market; travel distance was self-reported.

Data were analyzed using SPSS Statistics 23 (IBM Corp). Descriptive statistics were used to characterize survey responses, and logistic regression was used to assess the odds of PIUKP influencing fruit and vegetable shopping behaviors, adjusted for SNAP recipient status.

## Results

The survey was completed by 112 participants in year 1 and by 139 in year 2 (Table 1). In year 1, when no gas cards were offered, 16% reported receiving SNAP benefits. In year 2, when gas cards were offered, 30% reported receiving SNAP benefits.

**Table 1:** Association Between Exposure to Plate It Up Kentucky Proud Social Marketing Campaign and Farmers Market Purchases Among Residents of 6 Rural Kentucky Counties, 2015–2016

Characteristic	Year 1 (n = 112), %	Year 2 (n = 139), %
Preferred language is English	100	100
Female	77	88
Age, mean, y	49	51
Own car	87	85
Graduated from high school or completed general equivalency degree	24	25
Supplemental Nutrition Assistance Program recipient	16	30
White	97	96
Annual household income <$20,000	28	44
Consume whole fruit once per day or more	33	31
Consume green salad once per week or less	38	40
Consume other vegetables 2 or 3 times per week	34	37
Having recipe cards available at the market influenced purchase of fruits and vegetables	28	55
Having recipe sample available contributed to purchase of ingredients for the recipe sampled	36	54
Heard about the Plate It Up Kentucky Proud program in the past several weeks	69	50
Taking a food sample made you want to prepare that food at home	19	74

Participants who heard about PIUKP and took a food sample were 2.47 (95% confidence interval, 1.30–4.70) times as likely as those who had not heard about PIUKP to want to prepare the food item at home (Table 2). Awareness of PIUKP was not associated with recipe cards or food samples being available at the farmers market. 

**Table 2:** Logistic Regression Analysis^a^ of Association Between Exposure to Plate It Up Kentucky Proud and Farmers Market Shopping Behaviors Among Residents of 6 Rural Kentucky Counties, 2015–2016

Characteristic	Odds Ratio (95% Confidence Interval)	*P* Value
Does having recipe cards available at the market influence your buying of fruits and vegetables while at the market?	1.15 (0.56–2.30)	.70
Did having the recipe sample available contribute to your buying the ingredients for the recipe you sampled?	1.5 (0.79–3.00)	.21
If you took a food sample, did that food sample make you want to prepare that food at home?	2.47 (1.30–4.70)	.006

a Logistic regression model adjusted for the Supplemental Nutrition Assistance Program status; reference category is no.

## Discussion

In our study, shopping behaviors at rural farmers markets may have been influenced by the PIUKP campaign, as one possible mechanism for altering shopping patterns. Although awareness of PIUKP was not associated with the availability of recipe cards or food samples, awareness of PIUKP was associated with a willingness to prepare the recipe at home. Our findings are not consistent with previous research suggesting recipe samples, cooking ideas, and food demonstrations are ways to improve purchasing habits (8). Our study highlights how a community-based marketing campaign may be effective at influencing shopping behaviors of rural residents, especially when implemented over an extended period of time (our 2-year study period).

SNAP users frequently use electronic benefit transfer (EBT) to purchase foods. Because 30% of the participants in year 2 were SNAP users, it can be inferred that not having the ability to accept these benefits can be a barrier to shopping at farmers markets (9). EBT terminals may be especially important in rural areas, such as those observed in this study, where gas cards for SNAP participants, who frequently use EBT benefits, increased participation. Use of an EBT card facilitates shopping at farmers markets (10). Because farmers markets offer fresh, local fruits and vegetables, the US Department of Agriculture prioritized expanding access to these foods for SNAP recipients at farmers markets (11). The relationship between SNAP benefits and farmers market use warrants further study.

This study has the following limitations: its cross-sectional survey design (which prohibits us from drawing conclusions on cause and effect), a small sample size, and self-reporting of data. Also, the increase in awareness of PIUKP we observed may be because high percentage of individuals being aware of the PIUKP program may be related to communities conducting PIUKP events in other outlets (grocery stores and supercenters). Farmers market shoppers may also have been familiar with PIUKP in year 2 because they recalled it from year 1. Also, not all farmers markets accepted SNAP benefits, and 30% of study participants were SNAP recipients.

This study assessed the influence of PIUKP on fruit and vegetable shopping behaviors at rural farmers markets, and the results support the hypothesis that a community-based marketing campaign can increase rural farmers market patrons’ willingness to purchase fruits and vegetables. The study findings emphasize the importance of using community-based marketing strategies at farmers markets to increase the purchase of fruits and vegetables in rural communities. These findings may also be helpful for studies examining the influence of a similar program at grocery stores offering produce year-round in these communities.
